# Arguments Reinforcing the Three-Domain View of Diversified Cellular Life

**DOI:** 10.1155/2016/1851865

**Published:** 2016-12-05

**Authors:** Arshan Nasir, Kyung Mo Kim, Violette Da Cunha, Gustavo Caetano-Anollés

**Affiliations:** ^1^Department of Biosciences, COMSATS Institute of Information Technology, Islamabad, Pakistan; ^2^Biological Resource Center, Korea Research Institute of Bioscience and Biotechnology, Jeongeup, Republic of Korea; ^3^Division of Polar Life Sciences, Korea Polar Research Institute, Incheon, Republic of Korea; ^4^Institut Pasteur, Unité de Biologie Moléculaire du Gène chez les Extrêmophiles (BMGE), Département de Microbiologie, 75015 Paris, France; ^5^Evolutionary Bioinformatics Laboratory, Department of Crop Sciences, University of Illinois, Urbana, IL, USA

## Abstract

The archaeal ancestor scenario (AAS) for the origin of eukaryotes implies the emergence of a new kind of organism from the fusion of ancestral archaeal and bacterial cells. Equipped with this “chimeric” molecular arsenal, the resulting cell would gradually accumulate unique genes and develop the complex molecular machineries and cellular compartments that are hallmarks of modern eukaryotes. In this regard, proteins related to phagocytosis and cell movement should be present in the archaeal ancestor, thus identifying the recently described candidate archaeal phylum “Lokiarchaeota” as resembling a possible candidate ancestor of eukaryotes. Despite its appeal, AAS seems incompatible with the genomic, molecular, and biochemical differences that exist between Archaea and Eukarya. In particular, the distribution of conserved protein domain structures in the proteomes of cellular organisms and viruses appears hard to reconcile with the AAS. In addition, concerns related to taxon and character sampling, presupposing bacterial outgroups in phylogenies, and nonuniform effects of protein domain structure rearrangement and gain/loss in concatenated alignments of protein sequences cast further doubt on AAS-supporting phylogenies. Here, we evaluate AAS against the traditional “three-domain” world of cellular organisms and propose that the discovery of Lokiarchaeota could be better reconciled under the latter view, especially in light of several additional biological and technical considerations.

## 1. Introduction

The discovery of the novel candidate archaeal phylum “Lokiarchaeota” from metagenomic samples taken from sites near Loki's Castle hydrothermal vents of the Arctic Ocean was recently reported [1]. There are two interesting aspects to this discovery: (i) several eukaryotic signature proteins (ESPs) related to membrane remodeling, cell division, and the cytoskeleton, previously thought to be either absent or rare in akaryotes (Archaea and Bacteria;* sensu* [2]), were detected in the composite Lokiarchaeota genomes (Loki 1, Loki 2, and Loki 3), and (ii) phylogenomic analyses of concatenated alignment of 36 conserved proteins revealed that eukaryotes and Lokiarchaeota grouped together within Archaea, suggesting an* archaeal ancestor scenario* (AAS) for the origin of eukaryotes [3]. The AAS thus favors a two-domain (2D) view of the tree of life (ToL) where eukaryotes emerge from within Archaea, specifically as sister group to the proposed TACKL (including Thaumarchaeota, Aigarchaeota, Crenarchaeota, Korarchaeota, and Lokiarchaeota) superphylum [4, 5], after a likely merger of archaeal microbes (resembling Lokiarchaeota) and the mitochondrial ancestors [6].

AAS is fast becoming an accepted scenario to explain deep evolutionary history (e.g., [7–9]) and the origin of eukaryotic cells [10, 11]. Except for some dissenting opinions [12], Lokiarchaeota is now commonly viewed as the “missing link” in the transition from “simple” to “complex” life [1]. However, several key differences in the membrane biology, biochemistry, and virospheres of Archaea and Eukarya seem at odds with AAS (see [13] for a recent review). Simultaneous ToL reconstructions from concatenated ribosomal proteins and the small-subunit ribosomal RNA (SSU rRNA) gene produced conflicting topologies with the former supporting the AAS while the latter recovering the “Woesian” three-domain (3D) ToL [14] of cellular diversification into domains Archaea, Bacteria, and Eukarya [15]. Because protein sequences are generally more conserved than nucleic acid sequences, SSU rRNA genes possess relatively lower number of informative sites and a higher rate of evolution compared to concatenated ribosomal protein sets. SSU rRNA genes are therefore likely more sensitive to known issues such as the notorious long-branch-attraction (LBA) artifact [16]. In turn, ribosomal proteins exhibit strong compositional biases among the cellular domains of life that need to be better understood [15]. While the study provided an “updated” view of the ToL incorporating hundreds of uncultivated representatives of archaeal and bacterial genera (the so-called “microbial dark matter” [17]) into ToL reconstructions, the authors remained indecisive in picking either the 2D (from concatenated ribosomal proteins) or the 3D (from SSU rRNA) ToL to explain the origin of eukaryotes beyond any doubt [15]. The AAS is also in conflict with several historical phylogenetic and phylogenomic frameworks such as phylogenies built from SSU rRNA sequences [14], single-gene alignments of ancient paralogous genes [18, 19], gene content and order [20, 21], concatenated gene [22] and protein domain [23, 24] sets, and abundance combination and architecture of protein structural domains in modern genomes [23, 25, 26] that have consistently supported the 3D ToL despite disagreements on the location of the root of the ToL and the fact that most generated trees are unrooted [27–30].

It has been argued however that the use of “advanced” models of sequence evolution with relaxed assumptions of homogenous amino acid compositions of gene products across sites and branches is necessary to recover the origin of Eukarya from within Archaea (see [31] for a recent review). However, the presence of distant outgroups (e.g., bacterial ribosomal proteins that are quite divergent from archaeal-eukaryotic counterparts but are used to root the ToLs) and fast-evolving species (e.g., Nanoarchaeota [32] and* Methanopyrus kandleri* [33]) in datasets can make even these sophisticated methods prone to LBA, as shown by recent simulations [29] (see also [34]). Moreover, a concatenated (i.e., supermatrix) approach to phylogenetics, as applied by Spang et al. [1] to support AAS, could be problematic especially when member genes have independent evolutionary histories. Simulations have shown that concatenated gene sets can produce aberrant trees with high bootstrap (BS) support [35]. The approach is also susceptible to heterotachy (i.e., unequal evolutionary rates among genes in a concatenated set) [35, 36], which can complicate inferring deep evolutionary relationships and can introduce distortions to interdomain calculations, among other issues (see Section 5). In light of these considerations, here we examine the evidence supporting the 2D scenario for the diversification of cellular life, perform taxa and character manipulations to reanalyze the dataset of Spang et al. [1] that supported the Lokiarchaeota-Eukarya sisterhood, and consider several biological and technical issues that weaken the 2D in favor of the 3D ToL.

## 2. Eukaryotic Genomes Are More Complex Than Mere Archaea-Bacteria Genomic Chimeras

AAS remains popular due to the purported chimeric nature of eukaryotic genomes [5, 37]. For example, Guy et al. (2014) wrote, “*The apparent genomic chimerism in eukaryotic genomes is currently best explained by invoking a cellular fusion at the root of the eukaryotes that involves one archaeal and one or more bacterial components*” [3]. Indeed, eukaryotic genomes include many genes that have homologs in Archaea and Bacteria. Genes exhibiting bacterial affinity generally perform metabolic functions while those with archaeal affinity perform informational roles (i.e., DNA replication, transcription, and translation) [37], though exceptions to this “rule” exist (see [38] for a recent review). The proponents of AAS claim that chimerism in eukaryotic genomes is best explained by invoking the transformation of an archaeon (host cell) into a eukaryote by the engulfment of the bacterial ancestor of mitochondria [1]. Thus, a new kind of cell would originate from fusion between two different kinds of cells, a scenario contested to be biologically implausible (see [13] for a recent review).

A coarse-grained examination of eukaryotic genomes also indicates that chimerism is apparently an oversimplification. For example, in addition to Archaea-like and Bacteria-like genes, eukaryotic genomes house a significant number of viral genes and viral-like retrotransposable genetic elements that are likely remnants of ancient viral infections [39, 40]. This viral-like genetic material should therefore imply a “third” partner contributing towards genomic chimerism in eukaryotes. Under AAS, this new partner must invade the eukaryotic genome (or originate de novo) after the proposed fusion event because eukaryotic RNA and retrotranscribing virus families have hitherto not been described in Archaea (see Figure  1 in [41]). This poses a conceptual problem because modern RNA viruses are likely relics of ancient RNA viruses that played significant roles in evolutionary history, perhaps even contributing to the discovery of DNA [42]. Moreover, a substantial number of eukaryotic core genes lack any homologs in akaryotes and were believed to be present in the last common eukaryotic ancestor (up to 40% according to [43]). Remarkably, Eukarya-specific and viral-like genes quantitatively exceed Archaea/Bacteria-like genes in eukaryotic genomes and not all Bacteria-like genes descended from the mitochondrial ancestor (Section 3). At first glance, these observations suggest that the Archaea-Bacteria chimerism is not an a priori requirement to explain eukaryogenesis. Instead, it rather underestimates the distinctive and global nature of eukaryotic genomes.

## 3. AAS Is Not Supported by Protein Structure Data

**Figure 1:**
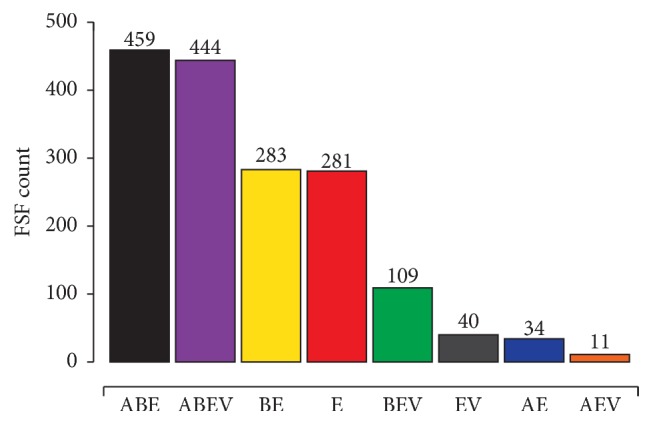
The global nature of eukaryotic proteomes. A total of 1,661 FSFs were detected by the SUPERFAMILY hidden Markov models [44, 45] in proteins coded by 383 completely sequenced proteomes of eukaryotes (FSF assignments of* Lokiarchaeum* were added a posteriori to data taken from [46]). Bars display the number of eukaryotic FSFs that either were shared with Archaea (A) and Bacteria (B) and viruses (V) or were unique to eukaryotes (E).

A dissection of the proteomic makeup of 383 completely sequenced eukaryal proteomes reveals the global nature of eukaryotic proteomes (Figure 1). A total of 1,661 protein domain fold superfamilies (FSFs) coded by eukaryotic proteomes can be divided into eight mutually exclusive groups: ABEV (universal), ABE (universal in cells), BEV (all except Archaea), AEV (all except Bacteria), AE (only in Archaea and Eukarya), BE (only in Bacteria and Eukarya), EV (only in Eukarya and viruses), and E (unique to eukaryotes) (Figure 1). FSFs, as defined by the Structural Classification of Proteins (SCOP) database [52, 53], are collections of distantly related protein domains that share recognizable structural and biochemical similarities indicative of divergence from ancestral domain structures. FSFs are thus highly conserved molecular characters that are useful tools to examine deep evolutionary relationships, especially because protein structure is more refractory to change compared to gene and protein sequences that are prone to mutational saturation over long evolutionary distances [54–56].

**Figure 2:**
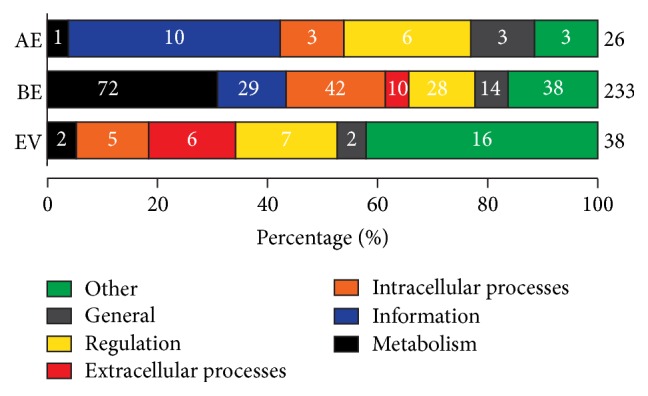
Functional composition of AE, BE, and EV Venn groups. FSFs were mapped to one of the seven major functional categories of molecular functions (i.e.,* Metabolism*,* Information*,* Regulation*,* Intracellular Processes*,* Extracellular Processes*,* General*, and* Other*), as defined by Christine Vogel (http://supfam.org/SUPERFAMILY/function.html) [47–49]. Numbers on the right indicate total number of FSFs for which functional annotation was available. Numbers on bars indicate total number of FSFs annotated to each of the seven major functional categories in that group.

The AE, BE, and EV groups are of particular interest to this discussion as they imply sharing of homologous FSFs in only two sets of proteomes. The numbers alone are interesting as there is an 8-fold difference in the number of eukaryotic FSFs shared only with Bacteria compared with those shared only with Archaea (283 BE versus 34 AE). This bias challenges both the AAS [1] and the traditionally accepted Archaea/Eukarya sisterhood [14], as one should expect greater sharing between Archaea and Eukarya under these models. Moreover, the EV group even outnumbers the AE FSFs (40 versus 34). While it has been argued that viruses frequently pickpocket cellular genes [57], this historical “belief” has been challenged by several large-scale bioinformatics explorations that suggest gene flow from viruses to cells in fact exceeds gene transfer in the opposite direction [46, 58, 59]. Viruses can also create new genes during intracellular replication using host cell machinery (e.g., ~70–80% of viral genes lack cellular homologs; see Figure  1 in [46]) and some of these genes can later be coopted by cellular genomes (refer to the “virocell” concept [60]). Indeed, 16 out of 38 (42%) EV FSFs perform* Other* functions, a functional category that includes proteins with either unknown or viral functions, suggesting they did not originate in Eukarya (Figure 2). Eukaryotic proteomes also encode a substantial number of unique FSFs (281, ~17% of total eukaryotic FSFs) that confirm that eukaryotic genomes are not mere chimeras of genes mixed from different sources but are more complex than anticipated under the AAS model. In fact, the* Lokiarchaeum* genome (Loki 1) adds only 10 new FSFs to the archaeal repertoire [12] suggesting that the “bridge” between Archaea and Eukarya remains wide, especially when inferring homology at protein structure level.

It can however be argued that the presence of the same FSF in two different sets of proteomes could be due to horizontal gene transfer (HGT) or convergent evolution. However, similar concerns are also applicable to BLAST-based inferences of homology, especially because top BLAST hits are not necessarily orthologous [61]. Importantly, convergent evolution of protein folds is extremely rare [62] because the protein backbone is formed by unique “fingerprint” designs achieved through interactions between amino acid side chains. Due to the direct evolutionary constraint to maintain the overall biochemical function of proteins, disruptions in the protein structural backbone are generally resisted for longer periods of evolutionary time [55, 56, 63]. Moreover, the odds of originating convergent “fingerprints” are very small [62] and there is no reason to suggest that protein structure is relatively more influenced by nonvertical evolution than gene sequences (please see [54] and the references therein). In fact, the recent expansion in the availability of deposited protein structures in structure databases (123, 273 structural entries in RCSB Protein Data Bank [64] as of October 5, 2016) offers the unique opportunity to revise life history using an alternative and likely more reliable set of molecular characters.

## 4. Protein Domain Fold Superfamilies (FSFs) Shared Only by Bacteria and Eukarya (BE) Are Not Restricted to Metabolic Roles

The endosymbiosis of the mitochondrial ancestor likely contributed many metabolic genes to modern eukaryotic genomes [65, 66] and could therefore influence the large size of the BE group (Figure 1). This prompted us to inspect the functional makeup of the AE, BE, and EV groups (Figure 2). Interestingly, BE was not restricted solely to metabolic FSFs but included an ensemble of informational, general, and other FSFs involved in intracellular and extracellular processes (Figure 2). In fact, metabolic FSFs constituted only 31% of BE FSFs (72 out of 233) highlighting the partial contribution of metabolism-inspired gene transfer and enzymatic recruitment to the composition of the BE group. Moreover, eukaryotes shared more informational FSFs with Bacteria than Archaea (29 versus 10). The data therefore suggest that mitochondrial endosymbiosis does not fully account for the large numerical difference in the sizes of BE and AE FSFs. Instead, Bacteria-like eukaryotic genes can alternatively be explained by a combination of (i) endosymbiosis in a protoeukaryotic ancestor (i.e., not an archaeon), (ii) recent HGTs between bacterial and eukaryotic species, and/or (iii) Bacteria-Eukarya sisterhood in an alternative topology of the 3D ToL [28, 67, 68], without the need to invoke the AAS. It is important to note that, despite several concerns and the use of methods that do not root ToLs (reviewed in [29]), the early origin of Bacteria is taken by default or as a fact under AAS and corresponding phylogenetic trees are rooted using bacterial outgroup sequences. This rooting is ad hoc and could be problematic because it ignores a large body of work challenging the “traditional” bacterial rooting of the ToL [28, 30]. In other words, Bacteria and Eukarya share a wide range of molecular (283 FSFs) and biochemical features (e.g., similar lipid membranes) indicating perhaps a more complex evolutionary history than that explained by chimerism or nonvertical evolution [28].

Similarly, Archaea-like genes in eukaryotes can be explained under the Woesian 3D scenario by invoking a sister group relationship between Archaea and Eukarya, a view historically supported by phylogenies rooted with many paralogous gene sequences [18, 19]. Notably, this topology also accounts for the presence of several ESPs that are scattered in various members of Archaea [1]. Other alternatives involve the origin of the three cellular domains from a complex ancestor of life [69, 70] followed by selective loss of Archaea-like eukaryotic genes in Bacteria and loss of Bacteria-like genes in Archaea (e.g., [71]). For example, the distribution of FSFs in Archaea, Bacteria, Eukarya, and viruses revealed the existence of a shared “universal” core comprising 54% of total FSFs (903 ABE and ABEV FSFs out of a total of 1,661) (Figure 1). The large size of the universal core favors the view that the last common ancestor of cells (and viruses) was already more complex than anticipated (see also [72, 73]). Hence, the differential loss of genes can also account for their absence in one of the three cellular domains of life, especially because many akaryotic species are believed to evolve via genome reduction [74–76]. In summary, even ignoring evidence from FSF distributions, alternative explanations can account for the purported chimerism that is at the root of AAS models suggesting that chimerism could be an oversimplified interpretation of eukaryotic genomes.

## 5. Technical Issues Related to Taxon and Character Sampling Question AAS

Next, we focus on the more technical aspects of the AAS. It is true that simple genomic comparisons, such as those of FSF distributions, are no substitutes to formal phylogenetic studies (though they have been supported by comparative and phylogenomic exercises [28]). As case study, we evaluated the technical design of the study of Spang et al. [1]. The authors recovered a clade of Lokiarchaeota and Eukarya from trees reconstructed from a concatenated alignment of 36 “universal” proteins in 104 taxa (84 Archaea, 10 Bacteria, and 10 Eukarya, hereafter the 84-10-10 dataset). We focus our discussion on two aspects of their tree reconstruction: (i) taxon sampling and (ii) the use of concatenated alignments (i.e., character sampling and assembly).

**Figure 3:**
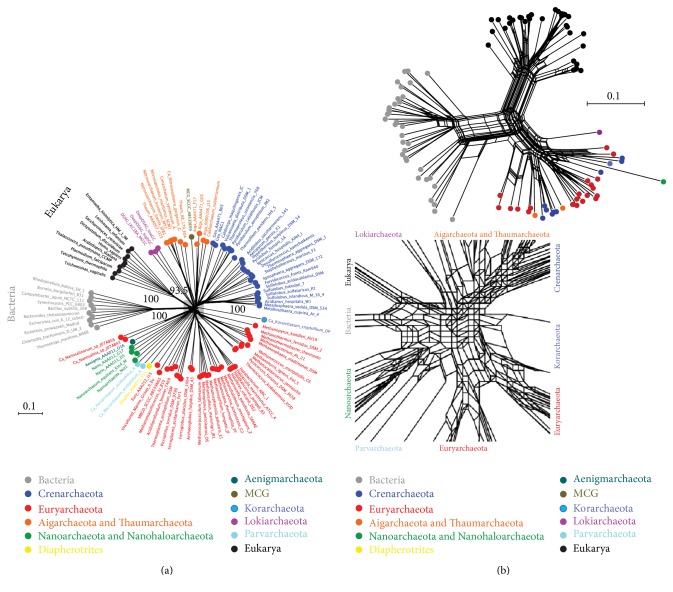
Distance networks do not support AAS. (a) The concatenated alignment of Spang et al. (2015) was provided by Lionel Guy and Thijs Ettema [1]. This alignment concatenated 36 genes (arCOGs) in 104 taxa (84-10-10 dataset) and was already trimmed by authors to remove sites containing >50% gaps. A splits-tree distance-based network (character sites = 10,547, LS fit = 99.97; *δ*-score = 0.25) reconstructed from the 84-10-10 dataset does not support AAS [1]. Eukaryotic proteomes are in close proximity to Lokiarchaeota but form a monophyletic group of their own. Numbers on branches indicate BS support values for deep split events. The inset shows reticulations at the base of the tree. MCG: miscellaneous crenarchaeotal group. (b) An unrooted splits-tree distance-based network (character sites = 493, LS fit = 99.61; *δ*-score = 0.24) reconstructed from 34-34-34 dataset sampled from Archaea (including* Lokiarchaeum*), Bacteria, and Eukarya and 493 characters corresponding to presence/absence of FSF domains in the universal ABE (459) and AE (34) groups of Figure 1. For this reconstruction, we only considered organisms exhibiting “free-living” lifestyles since parasitic and obligate parasitic organisms tend to have reduced genomes that are distorted by their holobiont relationship biasing the data matrix. The only “non-free-living” exception was Nanoarchaeota that was added to ensure consistency with the 84-10-10 dataset and to maximize the coverage of archaeal phyla [1]. Both unrooted networks reconstructed by SplitsTree (ver. 4.13.1) [50].

Taxon sampling is extremely important for the success of phylogenomic reconstructions as biased and uneven sampling can easily mislead evolutionary interpretations. As Delsuc et al. (2005) wrote,* “garbage in, garbage out”* [77], implying that even the best algorithms can produce false results when taxa/characters do not sufficiently represent extant biodiversity or are known to be problematic. First, overrepresentation of archaeal taxa and sparse selection of bacterial and eukaryal species (i.e., 84-10-10 in [1]) could be problematic, especially because the dataset includes several archaeal species that are sole members of their phylum (e.g.,* Candidatus* Korarchaeum cryptofilum), have unknown taxonomic affiliations (e.g., Nanoarchaeota [32, 78]), and/or are fast-evolving (Nanoarchaeota [32],* M. kandleri* [33]). Ideally, taxa should be sampled* randomly*,* equally*, and* densely* from each major group of organisms and increased for reliable tree reconstruction [79, 80] and fast-evolving members excluded [34, 81]. This is showcased by the basal positions of* M. kandleri* and* Thermotoga maritima* within the archaeal and bacterial subtrees in Spang et al.'s (2015) trees (Figure  2 in [1]).* M. kandleri* is a fast-evolving archaeon and its basal position in most phylogenetic trees is now considered a technical artifact [33, 82]. Similarly, the examination of slow-evolving sites in rRNA sequences has revised the phylogenetic placement of* T. maritima* [83] (see also [84]). To dissect these issues, we produced an unrooted distance-based phylogenomic network from the 84-10-10 Archaea-Bacteria-Eukarya concatenated sequence dataset [1]. Interestingly, the network did not group Eukarya within Archaea, recovering instead the 3D view of life (Figure 3(a)). Separately, we reconstructed distance networks from the occurrence (i.e., presence or absence) of universal FSFs (ABE) and FSFs shared by Archaea and Eukarya (34 AE) in 102 taxa sampled* randomly and equally* from the three cellular domains (i.e., 34 taxa each). Again, and despite the AE FSFs biasing reconstructions towards the AAS model, eukaryotes retained their unique identity and did not form a group within the archaeal subtree (Figure 3(b)).

**Figure 4:**
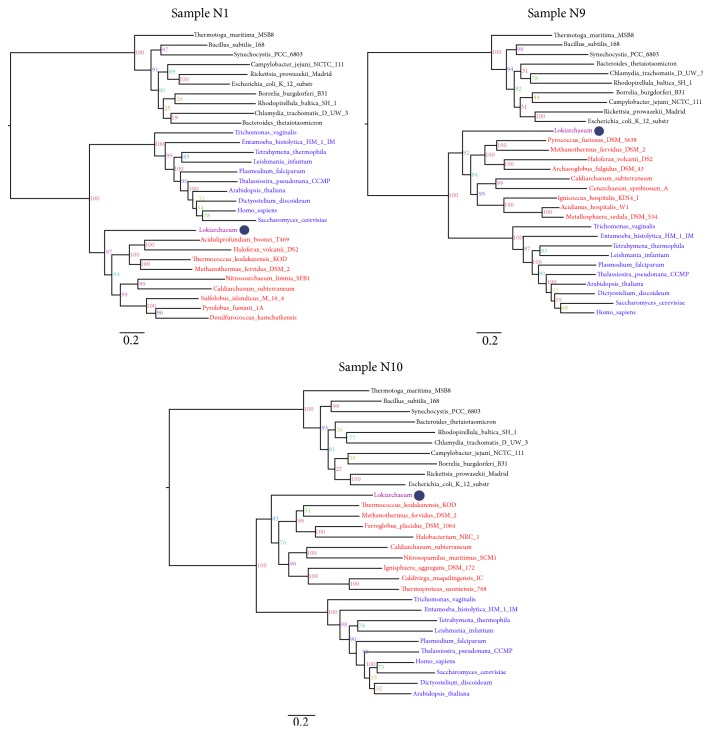
ML tree with revised datasets N1, N9, and N10 (see also Figures S1, S9, and S10 in the Supplementary Material available online at http://dx.doi.org/10.1155/2016/1851865). PhyML (ver. 3.1) [51] was used for ML tree reconstruction using LG amino acid substitution model and four categories of evolutionary rates (Γ4). The tree search topology operations were based on the BEST option (both NNI and SPR algorithms). Bacterial, eukaryal,* Lokiarchaeum* (Loki 1), and the rest of the archaeal species are indicated in black, blue, purple, and red, respectively. The purple circle identifies the position of Loki 1. The scale bar represents the average number of substitutions per site. Values at nodes represent support calculated by nonparametric bootstrap (out of 100).

While distance-based methods are no good substitutes to the sophisticated maximum likelihood (ML) and Bayesian analyses (used by Spang et al. [1]) that are less sensitive to LBA and account for relaxed assumptions of amino acid substitutions across sites and branches, they can be useful indicators of underlying conflicts between data and trees and can reveal the existence of reticulations [85]. Importantly, robust retrodictions should provide congruent reconstructions from parametric, nonparametric, and distance methods. Nevertheless, to test the impact of archaeal sampling on the robustness of tree topology, we repeated the phylogenetic analyses by producing 10 new datasets from the 84-10-10 dataset, sampling each time all 10 bacterial and eukaryal species but randomly extracting 10 archaea roughly representative of the known archaeal diversity (i.e., 3 Crenarchaeota, 3 Euryarchaeota, 1 Korarchaeota, 1 Aigarchaeota, 1 Thaumarchaeota, and 1 Lokiarchaeota; Figures S1–S10).* Lokiarchaeum* (Loki 1) was chosen as the Lokiarchaeota representative for these reconstructions. Despite using the same concatenated alignment of Spang et al. [1], balancing the number of taxa from each domain (i.e., the 10-10-10 datasets) had an immediate effect on the recovered phylogenies. In fact, 7 out of 10 reconstructed ML trees yielded monophyletic Archaea without any mixing of eukaryotic taxa (Figures S2–S8). For the remaining 3 trees that supported paraphyletic Archaea (Figures S1, S9, and S10), we observed that* M. kandleri* (a fast-evolving archaeon) was part of two reconstructions (Figures S9 and S10) indicating that this organism could distort tree topology. For the third tree that recovered paraphyletic Archaea (but in the absence of* M. kandleri*, Figure S1), we observed that group I euryarchaeotes (e.g., Thermococcales and Methanogens group I) were missing among the sampled archaeal taxa. Noticeably, Figure S5 that included* M. kandleri* but did not produce paraphyletic Archaea included both group I (i.e.,* Methanococcus maripaludis*, Methanococcales) and group II (*Ferroplasma acidiphilum*, Thermoplasmatales) euryarchaeotes confirming our initial observation that taxon sampling should be broad and inclusive of all groups with careful exclusion of fast-evolving species. Therefore, we produced 3 new phylogenies for the problematic datasets (i.e., Figures S1, S9, and S10) by replacing* M. kandleri* and* Candidatus* K. cryptofilum (the unique member of the putative phylum Korarchaeota, Figures S9 and S10) and* Cand*. K. cryptofilum and* Picrophilus torridus* (a group II euryarchaeote, Figure S1) by two sequences from group I Euryarchaeota (see trees in Figure 4). These revised datasets recovered the monophyly of Archaea (BS > 80%) and produced 3D ToLs (Figure 4). Our experimentation therefore hinted that the AAS (or 2D ToL) could perhaps be an outcome of including fast-evolving species and/or incomplete/unbalanced taxon sampling in phylogenetic datasets that could bias even the latest and sophisticated methods of tree reconstruction. Indeed, recent simulations have revealed that even Bayesian inferences could be prone to LBA when outgroups are too distant [29], a case, for example, when bacterial proteins are used to root ToLs. Indeed, separate ML and Bayesian reconstructions of DNA-dependent RNA polymerase (a universally conserved large protein and a reliable molecular marker [86]) performed after selecting 39 taxa each from Archaea, Bacteria, and Eukarya and after careful exclusion of fast-evolving archaeal species (*Nanoarchaea* and* M. kandleri*) recovered the 3D ToL and a sister relationship between Euryarchaeota and Lokiarchaeota (and its closest evolutionary relative Thorarchaeota [87]) indicating that the result obtained by Spang et al. [1] likely suffered from problematic experimental design (Da Cunha et al. ms. submitted). In summary, both distance-based and probabilistic methods of tree reconstruction and parsimonious inferences drawn from FSF distributions in eukaryotic proteomes challenge the phylogenetic reconstructions of Spang et al. [1] and the AAS model.

The second issue relates to the concatenated or supermatrix approach towards resolving deep evolutionary relationships. Spang et al. (2015) produced a concatenated alignment of 36 conserved genes in 104 taxa. This alignment was trimmed to remove sites with >50% gaps to filter out ambiguous regions. There could be two major problems with this approach: First, trimming using a 50% threshold (partial deletion) is highly dependent on the composition of inclusive taxa. Since the archaeal species dominated the dataset (i.e., 84 out of 104), a minimum of 32 archaeal species must possess the same* indel* present in all bacterial and eukaryal taxa to trim out ambiguous sites. The obvious problem with this approach is that one could trim out different regions when working with different datasets, as these vary in composition of Archaea, Bacteria, and Eukarya. While taxa deletion experiments of Spang et al. [1] claim to minimize the consequences of this issue, balancing the number of organisms sampled from each major group of organisms seems a logical* modus operandi*. Second, concatenated alignments are generally preferred because they yield greater resolution than single-gene markers and are relatively less susceptible to LBA (discussed in [77]). However, their use can be significantly compromised when the genes involved have different evolutionary histories [88], as Spang et al. (2015) themselves noted that the topologies of single-gene phylogenies (which were not shown) were* “often inconclusive with low support values at critical nodes”* [1]. In fact, only 5/36 genes in the concatenated alignment [1] supported the Lokiarchaeota/Eukarya affiliation. Thus, it becomes crucial to reconcile concatenated phylogenies against phylogenies of individual genes (that were included in concatenation) or to perhaps produce alignment-independent phylogenies to avoid these issues [54]. Indeed, several conflicts between concatenated gene sets and single-gene phylogenies specifically aimed towards resolving the phylogenetic relationship between Archaea and Eukarya have historically been reported (reviewed in [13]). To quote Forterre on this topic, “*One should be cautious in the interpretation of trees obtained from the concatenation of protein sequences that produce such contradictory individual trees”* [13]. It can also be a conceptual challenge to visualize the effects of protein domain gain, loss, inversions, and rearrangements in concatenation of several genes. These are well-known evolutionary processes influencing the history of molecular sequences [89] and could pose serious issues especially when primary sequence identity between proteins is very low, as could be the case when comparing distantly related taxa over long evolutionary timespans. Simulations have also shown that concatenated gene sets can lead to inconsistencies and produce misleading trees with high BS values [35], in addition to known issues of heterotachy [36].

Spang et al.'s [1] definition of “universal” proteins is also confusing since some bacterial and eukaryal taxa did not encode one or more of the 36 selected proteins. For example, 7 out of 10 eukaryal taxa did not include the Zn-dependent protease (arCOG04064) [1]. This shows that relatively little phylogenetic information (in terms of both taxa and character sampling) was contributed by bacterial and eukaryal sequences in their study. Moreover, because the dataset included a large number of ribosomal proteins (21 out of 36) that are quite divergent between Bacteria and Archaea/Eukarya, we suspect that the archaeal affiliation of eukaryotes was artificially enhanced under such experimental design (this would be true especially because trees were rooted using bacterial outgroup sequences). Finally, the authors detected several ESPs in the Lokiarchaeota genomes, claiming to be features unique to Lokiarchaeota and Eukarya. However, comparing FSF distributions across the three cellular domains of life and viruses indicates widespread presence of ESPs, especially in viruses (e.g., the Gelsolin-like domain superfamily [12]), suggesting perhaps that archaeal metagenomes were contaminated with eukaryoviruses. The authors also acknowledged the presence of “mimivirus” [90] in the metagenomic sample raising the possibility that its eukaryotic host could also be present. Even if the ESPs genuinely belong to Lokiarchaeota, they can still be explained by the Woesian 3D cellular world by considering a complex archaeal ancestor and subsequent gene loss in modern Archaea [38].

## 6. AAS Is at Odds with Biochemical and Virosphere Differences between Archaea and Eukarya

To quote Forterre, “*Generally speaking, it is very difficult to resolve ancient relationships by molecular phylogenetic methods for both practical and theoretical reasons, essentially because the informative signal is completely erased at long evolutionary distances*”… “*One possibility to bypass this phylogenetic impasse is to focus on biological plausibility*” [13]. AAS is especially weakened in this regard when one considers differences in the membrane biology and virospheres of Archaea and Eukarya. These issues have been raised before (e.g., [13, 38, 91–94]) but never satisfactorily addressed by the proponents of AAS. For example, transformation of one kind of cell into another has never been observed in nature even after known cases of HGT across domains (e.g., transfer of about 1,000 genes between Archaea and Bacteria [95]) and endosymbiosis events that are more “intimate” associations between cells but do not produce a new domain of life (e.g., plants remain eukaryotes despite acquiring about one-fifth of their genes from cyanobacteria [96]). Moreover, transformation of an archaeon into a eukaryote would imply transforming archaeal membrane lipids (ether-linked) into bacteria/eukarya like membrane lipids (ester-linked) for which there is no evolutionary rationale. Instead, the difference between the membrane biologies of Archaea and Bacteria/Eukarya could be taken as a powerful synapomorphy supporting the archaeal rooting of the 3D ToL [28]. Moreover, the complex makeup of eukaryotic cells differs greatly from the streamlined makeup of both Bacteria and especially Archaea (please note the substantial number of E FSFs in Figure 1). This gap is only marginally reduced by addition of the* Lokiarchaeum* genome that only adds 10 new FSFs to Archaea [12]. The scenario also seems logically incompatible because of little or no overlap in the genetics and morphology of archaeoviruses and eukaryoviruses (discussed elsewhere [97]). Specifically, many families of RNA viruses that infect eukaryotes seemingly cannot carry out a productive infection cycle in Archaea (though the archaeal virosphere remains largely unexplored [98]). Based on current data, under AAS, one should therefore postulate the late origin of eukaryotic RNA viruses after the transformation had taken place, as claimed by [99]. But this goes against several lines of evidence suggesting that RNA viruses originated very early in evolution and likely led the transition to a DNA world via retrotranscription [42], including a global phylogenomic study of cellular and viral proteomes [46]. The recent discovery of possibly multicellular eukaryotic fossils in 2.1-billion-year-old sediments pushes back in time the last common eukaryotic ancestor [100], further weakening the argument enforcing eukaryotic origins from within Archaea (reviewed in [13]). In short, AAS seems biologically implausible in light of several biological considerations.

## 7. Conclusions

Metagenomic explorations, development of single-cell sequencing technologies, and improvements in in silico reconstruction of (meta)genomes are yielding novel insights into our understanding of the evolutionary history of cellular organisms. The recent sequencing of Lokiarchaeota composite genomes and resulting phylogenetic analysis suggested an archaeal origin for the eukaryotic cell. The discovery has been widely publicized and the debate surrounding the origin of eukaryotes now considered by many to be settled. However, history inferred from protein structure data reveals a more global picture of the genetic composition of eukaryotic proteomes. Specifically, it takes into account the shared genes with Archaea, Bacteria, and viruses and challenges the purported eukaryotic genomic chimerism that is at the root of AAS models. While some interpret genomic chimerism in eukaryotes by invoking a fusion event at the root of eukaryote evolution, inferences redrawn from phylogenomic analyses performed after balanced taxon and character sampling, removal of fast-evolving species, and comparative analysis of protein structure distribution contradict that interpretation. Moreover, several biological and technical considerations are at odds with the proposed Lokiarchaeota-Eukarya phylogenetic affiliation and suggest that the 3D ToL may still be the more reasonable evolutionary scenario considering biological plausibility and support from molecular data.

## Supplementary Material

Figures S1-S10 represent 10 ML trees each consisting of a total of 30 taxa (10 Archaea, 10 Bacteria, and 10 Eukarya) from the 84-10-10 dataset of Spang et al. (A. Spang, J. H. Saw, S. L. Jørgensen et al., “Complex archaea that bridge the gap between prokaryotes and eukaryotes,” Nature, vol. 521, no. 7551, pp. 173–179, 2015). The trees test whether over-representation of archaeal taxa in (A. Spang, J. H. Saw, S. L. Jørgensen et al., “Complex archaea that bridge the gap between prokaryotes and eukaryotes,” Nature, vol. 521, no. 7551, pp. 173–179, 2015) could have favored the AAS.

## Additional Points

The concatenated trimmed alignments for 10-10-10 subsample trees can be downloaded at http://clustomcloud.kopri.re.kr/archaea/Trimmed_alignments_10_10_10.zip.
