# Supplementary material for: Arguments Reinforcing the Three-Domain View of Diversified Cellular Life
Source: Archaea. 2016 Dec 5;2016:1851865. doi: 10.1155/2016/1851865 (PMC5165138; doi:10.1155/2016/1851865)

**(The concatenated trimmed alignments for 10-10-10 subsample trees can**

**be downloaded at:**

**http://clustomcloud.kopri.re.kr/archaea/Trimmed_alignments_10_10_10.zip).**

**Supplementary Materials**

**Figures S1-S10.** A total of 10 ML trees were reconstructed representing 10 new datasets extracted from the 84-10-10 concatenated dataset of Spang et al. [1]. Each new dataset included 10 archaeal (randomly extracted from the 84 Archaea included by Spang et al. [1]), 10 bacterial (all part of [1]), and 10 eukaryal (all part of [1]) species. The composition of archaeal species varied in each of the datasets but corresponded to 3 Crenarchaeota, 3 Euryarchaeota, 1 Korarchaeota, 1 Aigarchaeota, 1 Thaumarchaeota, and 1 Lokiarchaeota (Loki 1 chosen as representative) to roughly represent known archaeal diversity. PhyML (ver. 3.1) [101] was used for ML tree reconstruction using LG amino-acid substitution model and four categories of evolutionary rates (Γ4). The tree search topology operations were based on the BEST option (both NNI and SPR algorithms). Bacterial, eukaryal, *Lokiarchaeum* (Loki 1), and the rest of archaeal species are indicated in black, blue, purple, and red, respectively. The purple circle identifies the position of Loki 1 in each tree. The scale-bar represents the average number of substitutions per site. Values at nodes represent support calculated by nonparametric bootstrap (out of 100).

**
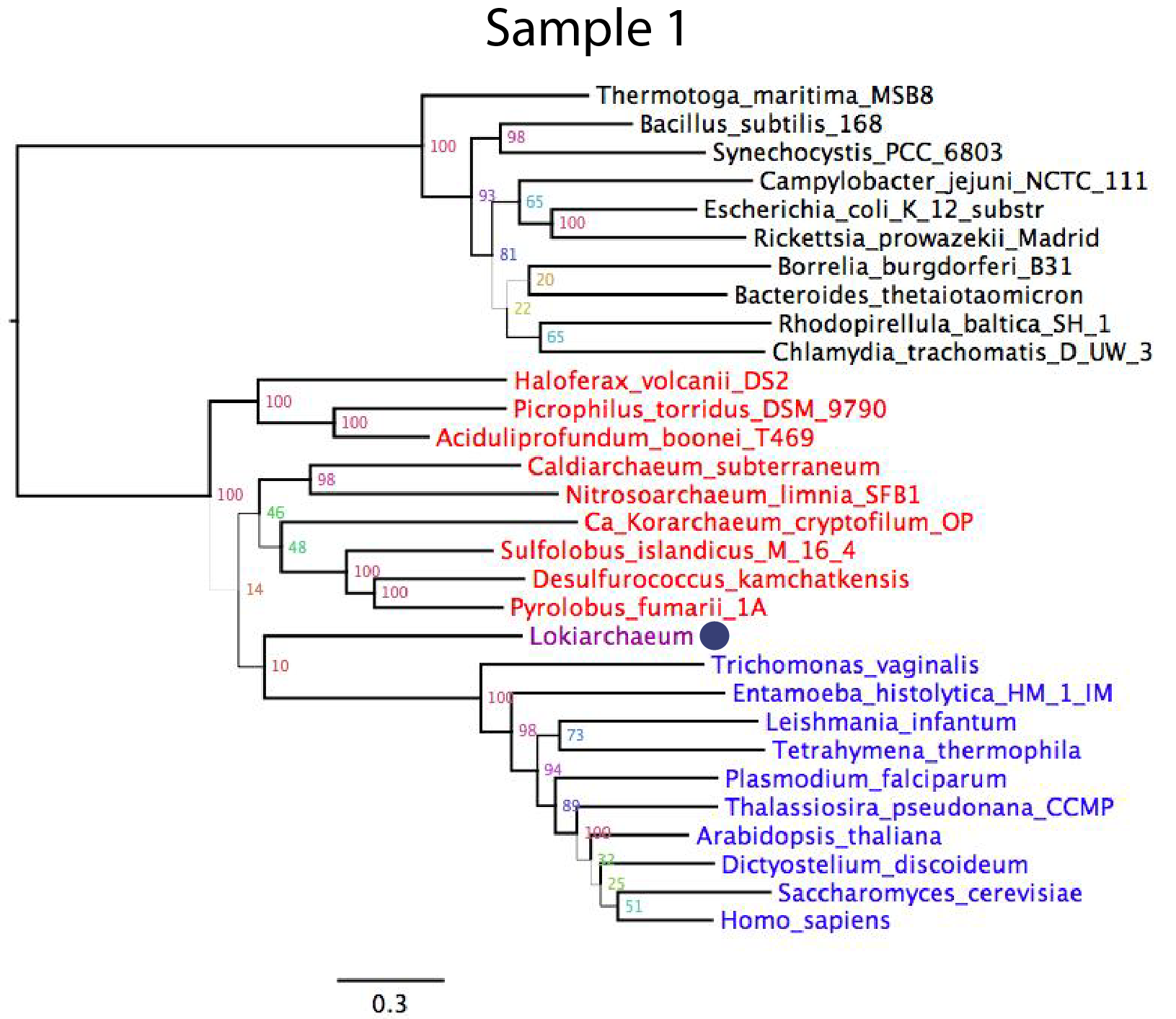
**

**
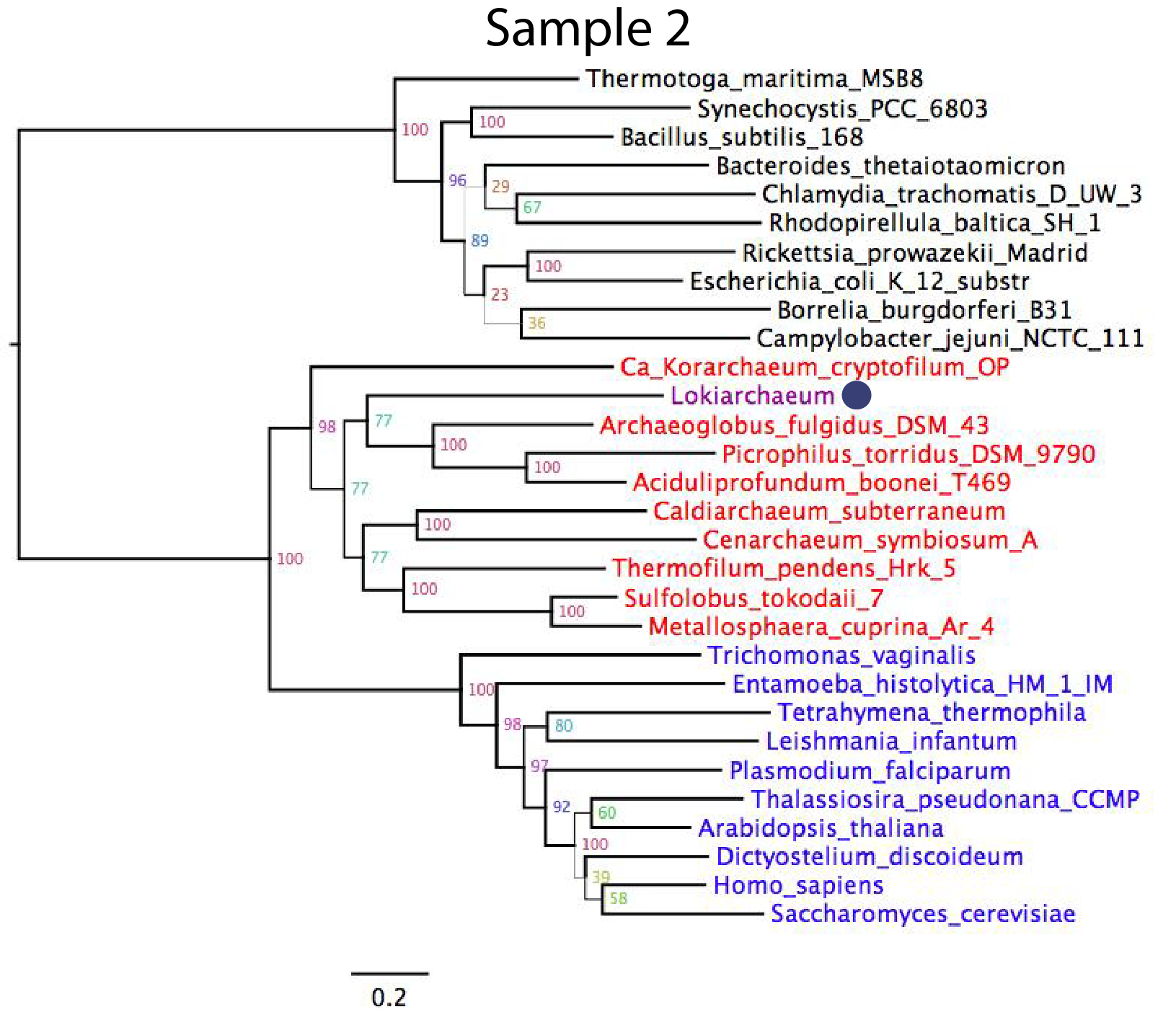
**


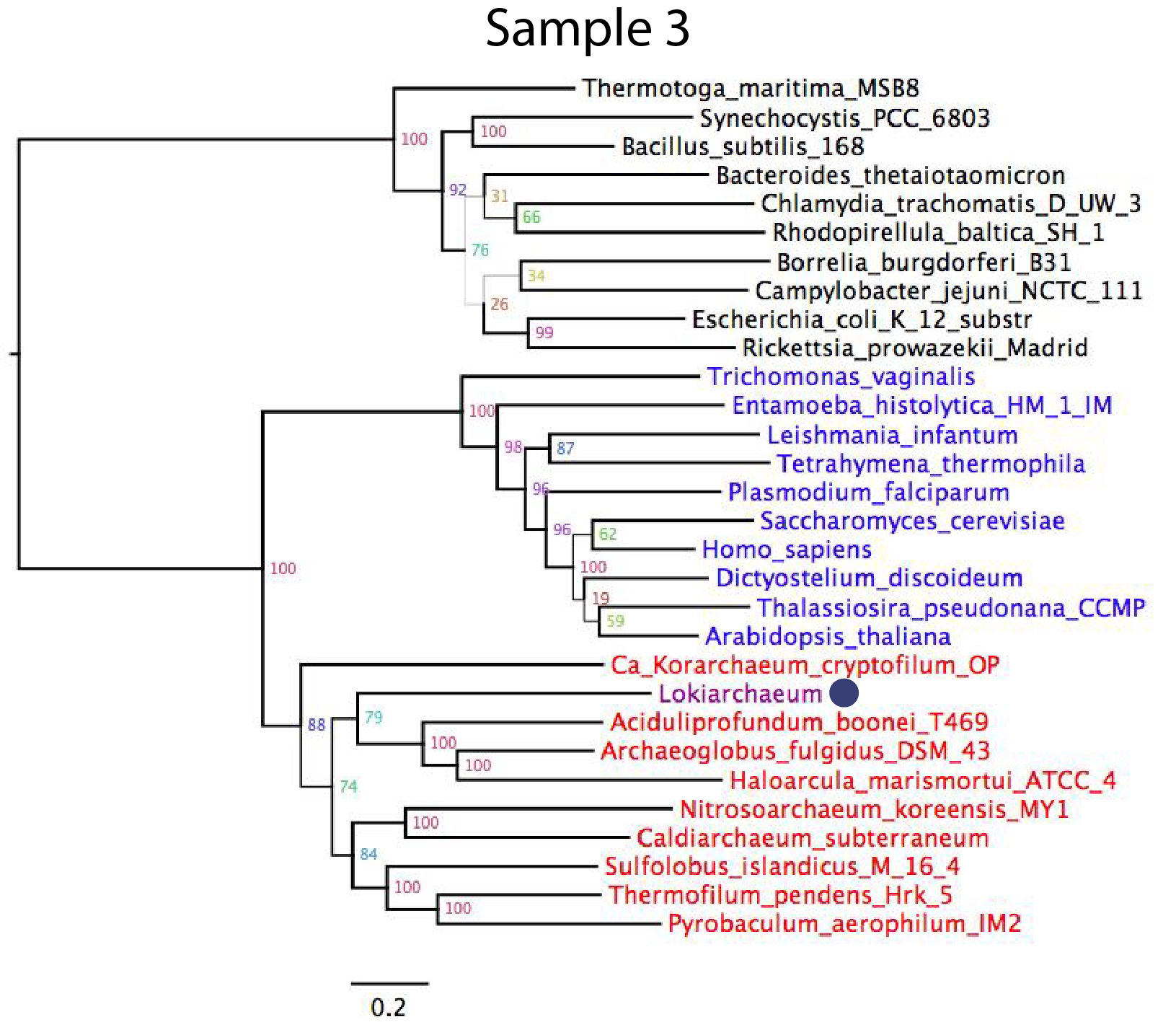


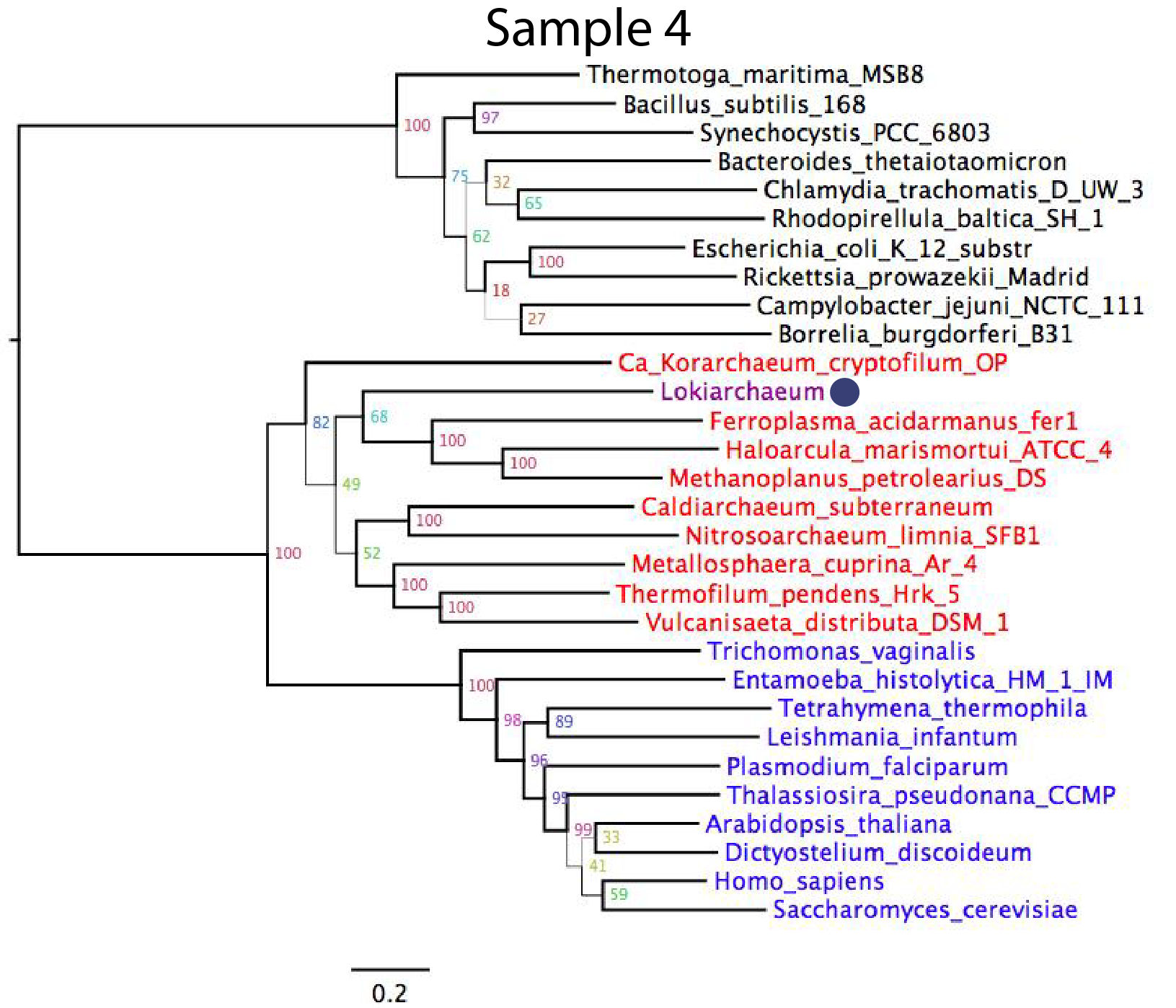


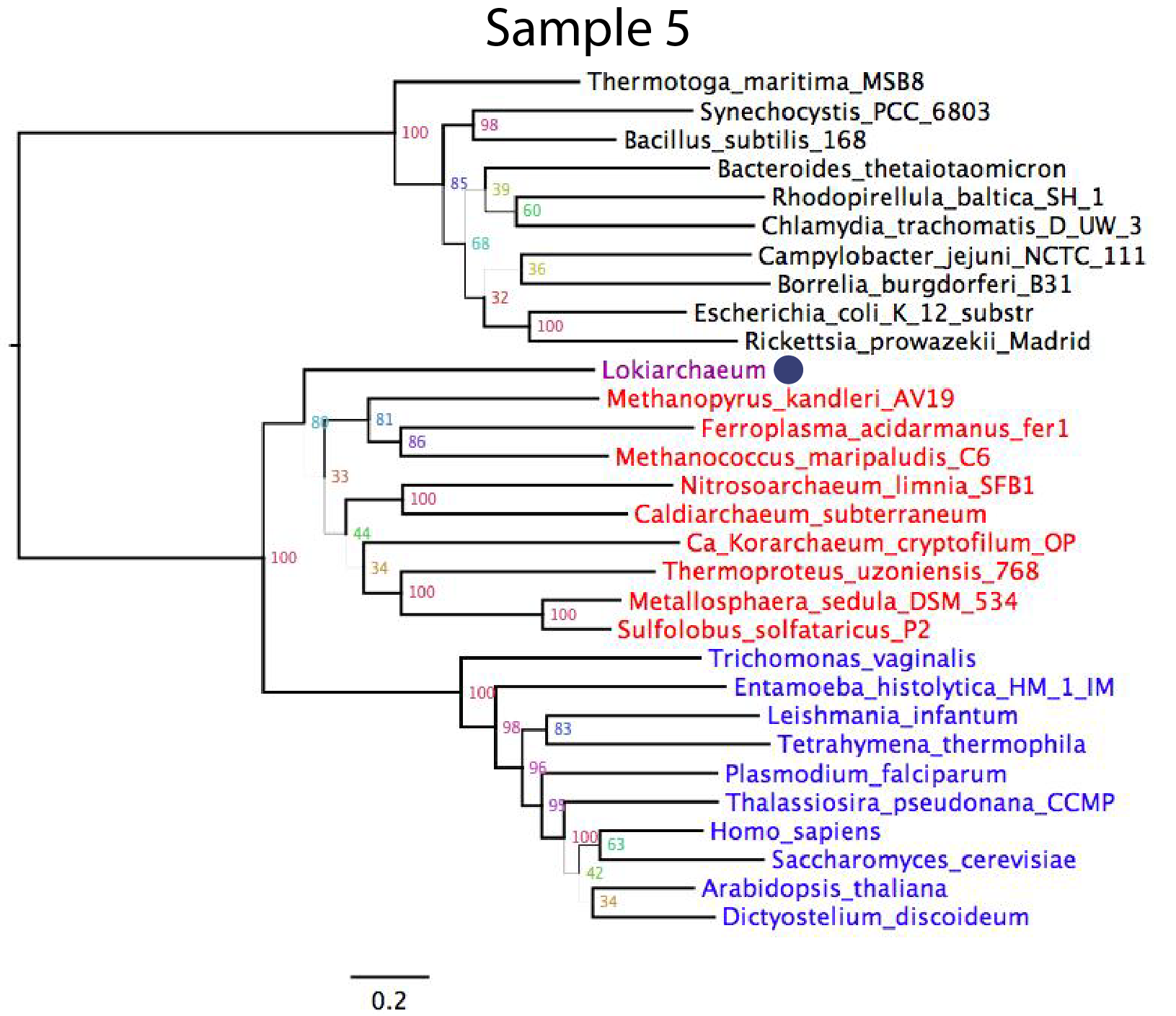


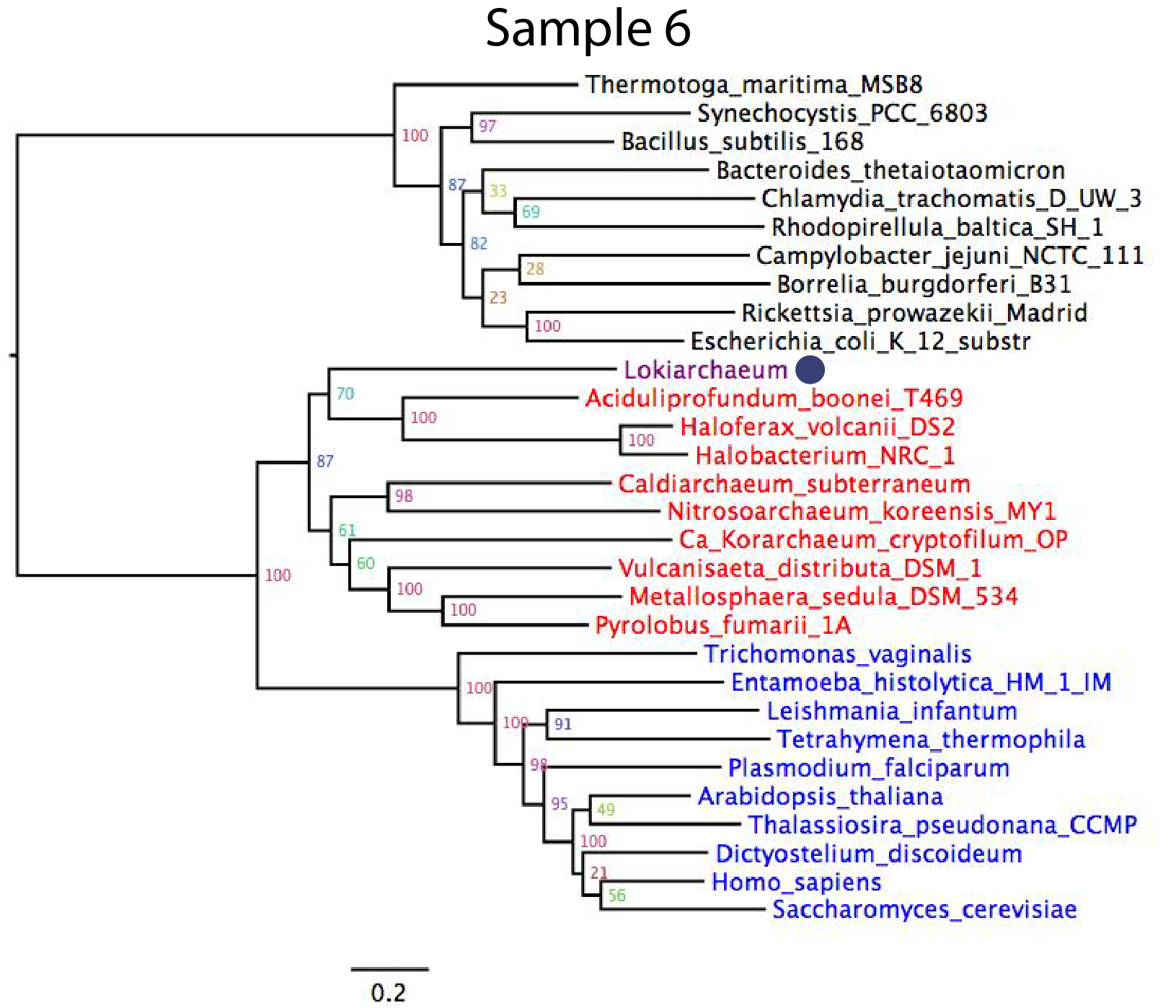


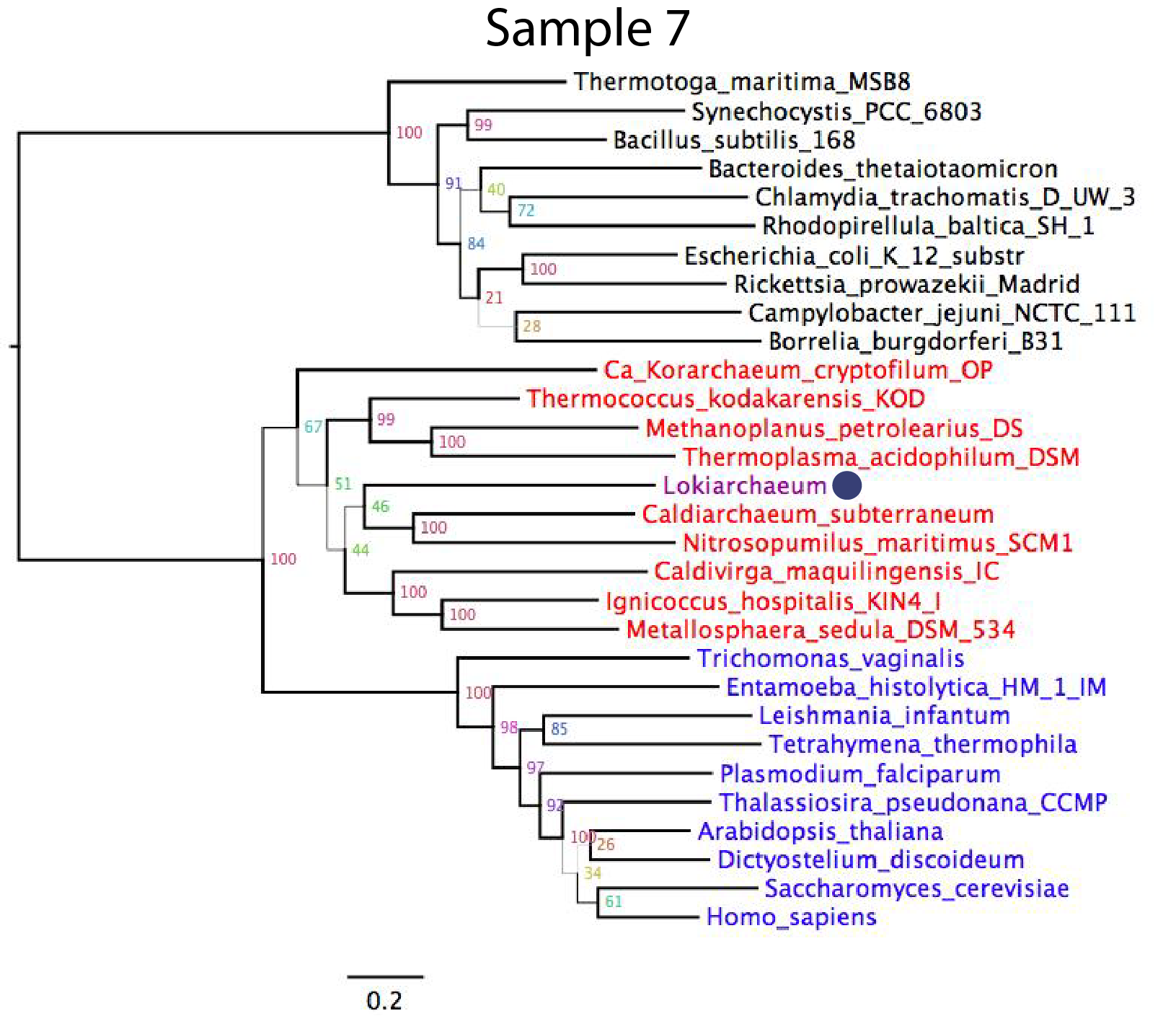


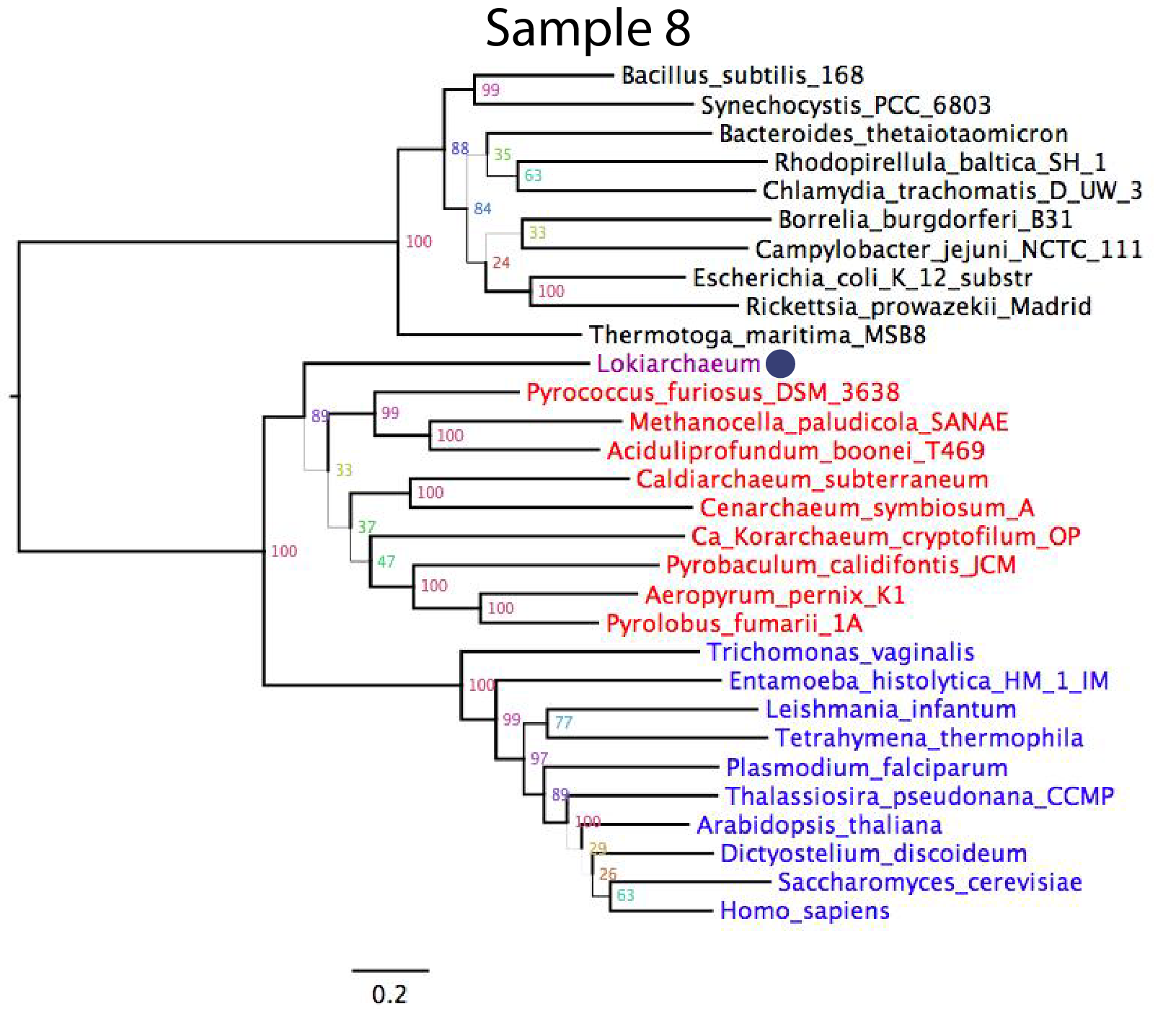


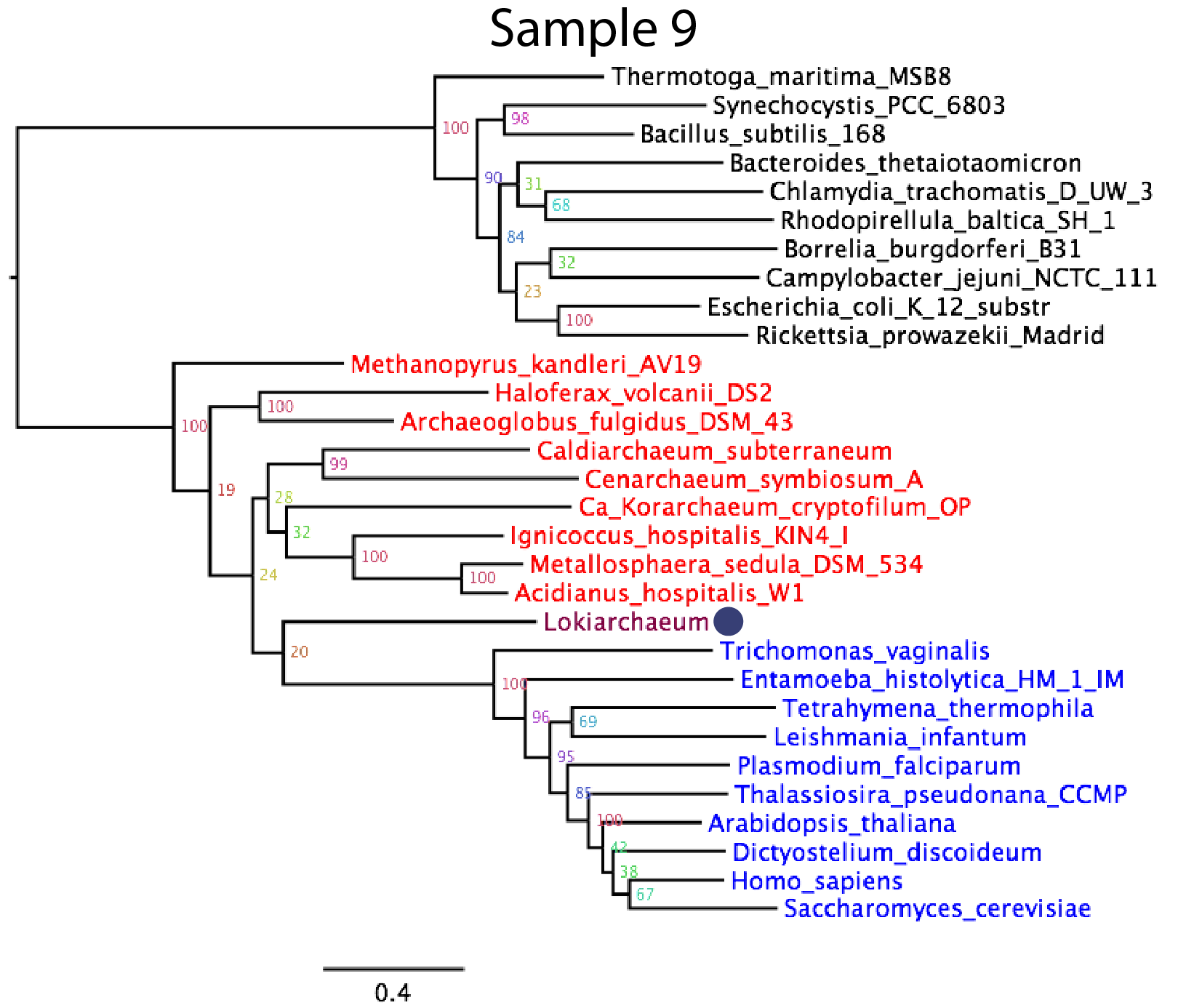


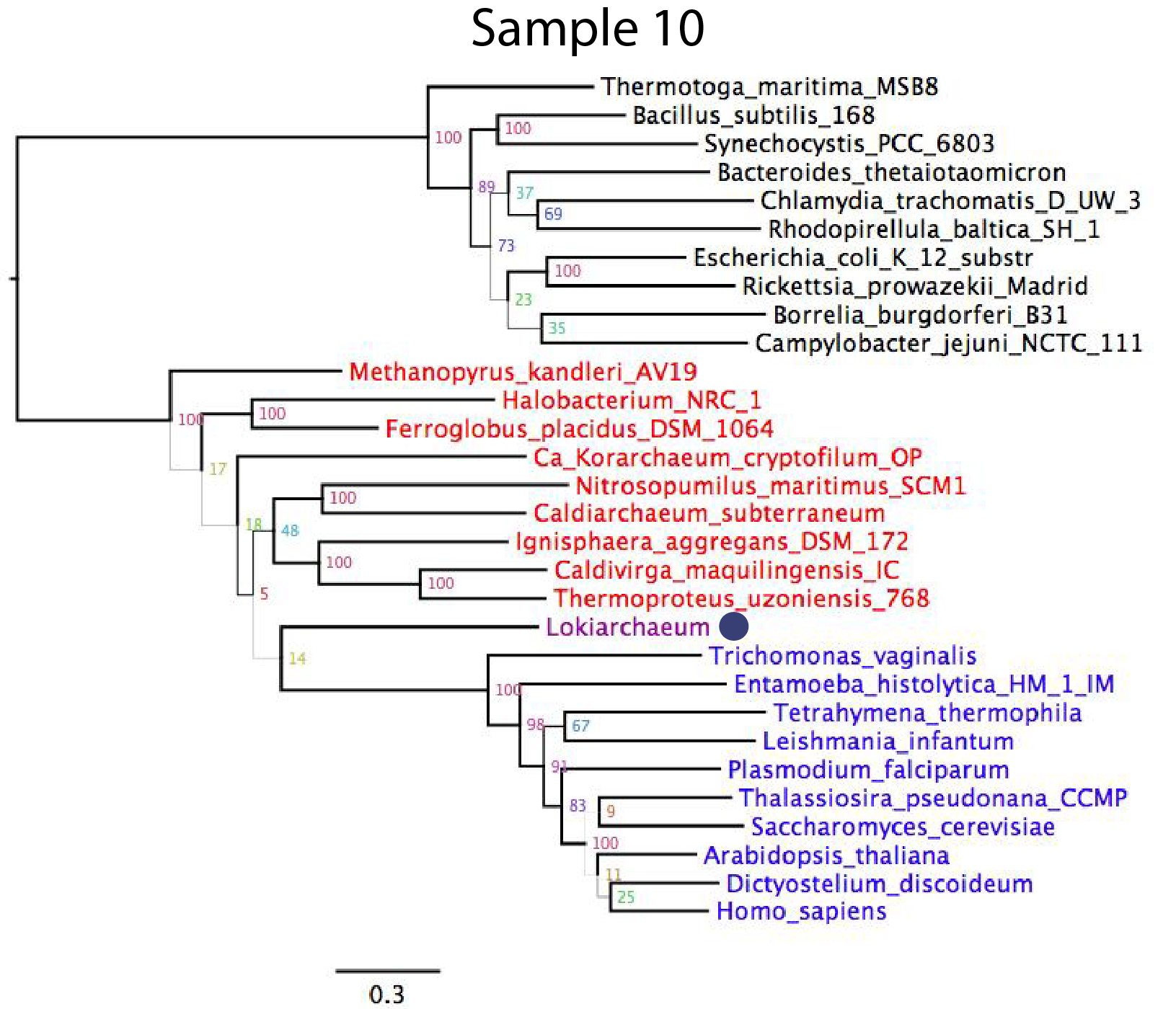

Supplement: Supplementary file 1 — Figures S1-S10 represent 10 ML trees each consisting of a total of 30 taxa (10 Archaea, 10 Bacteria, and 10 Eukarya) from the 84-10-10 dataset of Spang et al. (A. Spang, J. H. Saw, S. L. Jørgensen et al., “Complex archaea that bridge the gap between prokaryotes and eukaryotes,” Nature, vol. 521, no. 7551, pp. 173–179, 2015). The trees test whether over-representation of archaeal taxa in (A. Spang, J. H. Saw, S. L. Jørgensen et al., “Complex archaea that bridge the gap between prokaryotes and eukaryotes,” Nature, vol. 521, no. 7551, pp. 173–179, 2015) could have favored the AAS. [file 1851865.f1.docx]
